# The Extended Oxygen Window Concept for Programming Saturation Decompressions Using Air and Nitrox

**DOI:** 10.1371/journal.pone.0130835

**Published:** 2015-06-25

**Authors:** Jacek Kot, Zdzislaw Sicko, Tadeusz Doboszynski

**Affiliations:** National Centre for Hyperbaric Medicine, Institute of Maritime and Tropical Medicine, Medical University of Gdansk, Powstania Styczniowego, Gdynia, Poland; University of Bari, ITALY

## Abstract

Saturation decompression is a physiological process of transition from one steady state, full saturation with inert gas at pressure, to another one: standard conditions at surface. It is defined by the borderline condition for time spent at a particular depth (pressure) and inert gas in the breathing mixture (nitrogen, helium). It is a delicate and long lasting process during which single milliliters of inert gas are eliminated every minute, and any disturbance can lead to the creation of gas bubbles leading to decompression sickness (DCS). Most operational procedures rely on experimentally found parameters describing a continuous slow decompression rate. In Poland, the system for programming of continuous decompression after saturation with compressed air and nitrox has been developed as based on the concept of the Extended Oxygen Window (EOW). EOW mainly depends on the physiology of the metabolic oxygen window—also called inherent unsaturation or partial pressure vacancy—but also on metabolism of carbon dioxide, the existence of water vapor, as well as tissue tension. Initially, ambient pressure can be reduced at a higher rate allowing the elimination of inert gas from faster compartments using the EOW concept, and maximum outflow of nitrogen. Then, keeping a driving force for long decompression not exceeding the EOW allows optimal elimination of nitrogen from the limiting compartment with half-time of 360 min. The model has been theoretically verified through its application for estimation of risk of decompression sickness in published systems of air and nitrox saturation decompressions, where DCS cases were observed. Clear dose-reaction relation exists, and this confirms that any supersaturation over the EOW creates a risk for DCS. Using the concept of the EOW, 76 man-decompressions were conducted after air and nitrox saturations in depth range between 18 and 45 meters with no single case of DCS. In summary, the EOW concept describes physiology of decompression after saturation with nitrogen-based breathing mixtures.

## Introduction

Saturation diving is defined as the situation where one is at depth or pressure for a long enough period of time to have the partial pressures of the dissolved gas in the body at equilibrium with the partial pressure of those in the ambient atmosphere [1]. The minimum time for reaching saturation depends on the slowest compartment of the human body, which dissolves inert gas. The saturation process and the rate of change in partial pressure depends on the difference between partial pressures of inert gas in an inhaled breathing mixture and dissolved in a body compartment. So, mathematically, it is described by the half-time of the exponential process, defined as the time it takes for the compartment to take up 50% of the difference in dissolved gas capacity at a changed partial pressure. Practically, after six half-times, every compartment is almost fully saturated (98.44%) [2]. From this point, decompression after exposure is the longest one and no longer depends on time at pressure.

From the physical point of view, decompression after saturation is a transition from one steady state—saturation at pressure—to another one: saturation at surface. It is defined by the borderline condition for time spent at a particular depth (pressure) and breathing gas.

Saturation decompression is a delicate physiological process. In the human body (assuming standard volume of approximately 80 liters of tissues), there is about 1.37 L of nitrogen (N2) dissolved at atmospheric conditions where partial pressure of nitrogen is 0.79 ata [3]. It means that after saturation exposure using compressed air at 18 m (2.8 ata) there is approximately 2.5 L of nitrogen dissolved in tissues in excess to surface conditions, which must be eliminated gradually during decompression. Standard decompression time after saturation exposure with compressed air at a plateau depth of 18 m (2.8 ata) is about 36 hours [4], which gives the average elimination rate of nitrogen about 1.2 ml/min. This is a low value when compared to other physiological gas exchanges such as the metabolic turn-over of oxygen to carbon dioxide, when about 250 ml of oxygen is consumed each minute to produce about 200 ml of carbon dioxide. Moreover, saturation decompression is a long-lasting process. For nitrogen based gas mixtures, it takes about 18 hours to decompress by 10 meters of depth. Any violation of the physiological desaturation process at the beginning of decompression can lead to the creation of gas bubbles and symptoms of decompression sickness (DCS) many hours later, regardless of whether the diver is still under pressure or has already surfaced [5, 6].

For operational purposes, safety of saturation decompression can be easily achieved by slowing down pressure reduction. Such experimental prolongation of decompression had already been used in the past. In 1984, Thalmann reported a development of 18 m (60 fsw) air saturation decompression schedule [7]. Initially, he tried to decompress divers after air saturation using a heliox decompression rate (4 ft/hr to 50 fsw, 3ft/hr to 0 fsw, which gives about 21 hours), but failed, due to the high rate of DCS (four out of ten divers). In a subsequent seven series of 10 or 11 divers, the decompression rate gradually decreased with a reduced rate of DCS until the last series, when no DCS was observed in a group of 10 divers. This last schedule (3 ft/hr from 60 to 40 fsw, 2 ft/hr from 40 to 20 fsw and 1 ft/hr from 20 fsw to surface, which gives about 33 hours in total) had been accepted and, in fact, after extension by additional time spent at 4 fsw to total of 36 hours, is still binding as Treatment Table 7 [4]. A similar approach was used during extremely deep dives to 650 and 686 meters of the ATLANTIS series, when DCS already occurred during decompression [8, 9]. After stopping decompression and slight recompression for the treatment of symptoms, further decompression was commenced at an empirically decreased rate. In both examples, an empirical reduction of decompression rates solved the problem of DCS, but did not lead to an explanation of the physiological process behind it.

Despite more than 100 years of extensive research on decompression, we still lack a uniform theory of decompression, which could be used for all types of diving, including deep bounce dives, repetitive dives, and saturation dives. There is no possibility of extrapolating data from non-saturation dives to saturation ones. In 1997, when the US Navy released “new” air decompression tables, Survanshi et al reported that in “old” US Navy tables developed in 1956 and tested with 568 single man-dives and 62 double repetitive-dives, the probability of DCS increased significantly with longer decompression times; and for decompressions lasting longer than 6 hours, it had been accepted to expect 18–36% of DCS [10]. Still, there is a long way to go to saturation decompressions lasting several days. Since then, those decompression tables have been modified several times, but there are always time limits at every depth, from which a dive should be treated as an “exceptional” dive with a higher expected risk of DCS [4]. There is no single decompression system published where saturation decompression is treated the same way as a non-saturation bounce dive; even if from a physiological point of view, there should be no difference.

Nevertheless, the trial and error approach has led to a successful definition of operational procedures, and has practically “eradicated” DCS from saturation exposures, which moved diving companies away from research on physiology of saturation and decompression. However, we recently observed that some large organizations are reviving studies in this field [11] (M. Gennser, personal communication). Therefore, **we decided to present the complete set of data for the first time, which had been used for the creation of the Polish system of air and nitrox saturation decompressions using the novel concept of the Extended Oxygen Window**. It started in the 1980s, but it is still actual and valid, and has never been published *in extenso*.

## Model Description

After any exposure, the tissue tension (p) of any inert gas at an absolute pressure (P) is simply calculated on the basis that the rate of uptake or elimination of gas by any tissue is proportional to the driving force ΔP, equal to (P-p)—Eq 1.

$$\frac{dp}{dt}=k\cdot (P-p)$$ (1)

where k is the proportionality constant that embodies the effective resistance of that tissue to gas exchange with circulating blood, but is often expressed as a time to half saturation (half-time or T1/2) (Eq 2)[12].

$$k=\frac{\text{ln}(2)}{T_{1/2}}$$ (2)

In their pioneering publication, Haldane et al. advocated using five hypothetical “tissues” with half-times of 5, 10, 20, 40 and 75 minutes to be fairly representative of a continuous spectrum of response time [2]. Moreover, he suggested that after any exposure (including saturation), ambient pressure can be safely halved, and such pressure reduction should not induce DCS. Later on, many modifications to those assumptions were introduced, including an increase in the number of compartments, values of their half-times, maximum allowable pressure reduction after exposure, and differentiation of this pressure reduction between compartments. (For a good review on the development of decompression models, we refer the reader to other studies [13].)

Theoretically, from the point of view of saturation decompressions, when all body compartments are fully saturated, the number of parameters that sufficiently describe the elimination of inert gases can be reduced to only two: the maximum allowable gradient for elimination of inert gas ^max^ΔP, and a half-time of the limiting compartment (slowest tissue) for specific inert gas (nitrogen or helium), which limits inert gas elimination (^max^T1/2). If we assume that the model should decrease ambient pressure at the same rate that inert gas is eliminated from the slowest (limiting) compartment, the decompression rate can be described as below (Eq 3).

$$rate=\frac{\text{ln}(2)}{T_{1/2}}\cdot \Delta P$$ (3)

In the past, when creating the Polish system of saturation decompressions, we assumed that both parameters (^max^ΔP and ^max^T1/2) must be based on physiological values. In summary, the model consists of the following assumptions:

1. Inert gas desaturation from the human body fully saturated at a given pressure is a continuous process limited by perfusion.
2. The maximum allowable rate of pressure reduction (decompression rate or dp/dt) depends proportionally on the maximum persistently allowable elimination gradient from the limiting compartment (^max^ΔP) and on the elimination coefficient specific for this compartment (mink), which in turn is inversely proportional to the longest tissue half-time (^max^T1/2), according to the following equations:

$$rate^{\text{max}}=k^{\text{min}}\cdot \Delta P^{\text{max}}$$ (4)

$$k^{\text{min}}=\frac{\text{ln}(2)}{T_{1/2}^{\text{max}}}$$ (5)

1. The maximum persistently allowable elimination gradient from the limiting compartment (^max^ΔP) corresponds to the Extended Oxygen Window (EOW), which in practice equals to partial pressure of oxygen (PiO2) in inspired breathing mixture according to the following equation:

$$\Delta P^{\text{max}}=PiO_{2}$$ (6)

1. Tissue half-time for elimination of nitrogen from the limiting compartment (^max^T1/2) is 360 min, and calculated value of k (minkN2) is 0.001925 min-1 [14, 15].
2. At the beginning of saturation decompression, there is a partial unsaturation in tissues, which allows a faster initial rate of decompression. The value of this unsaturation equals to the EOW, which in turn equals to partial pressure of inspired oxygen (PiO2). The decompression rate during passing of the EOW can be safely increased as it depends on the elimination rate of inert gas from compartments with half-times shorter than 360 min.

**Fig 1:**
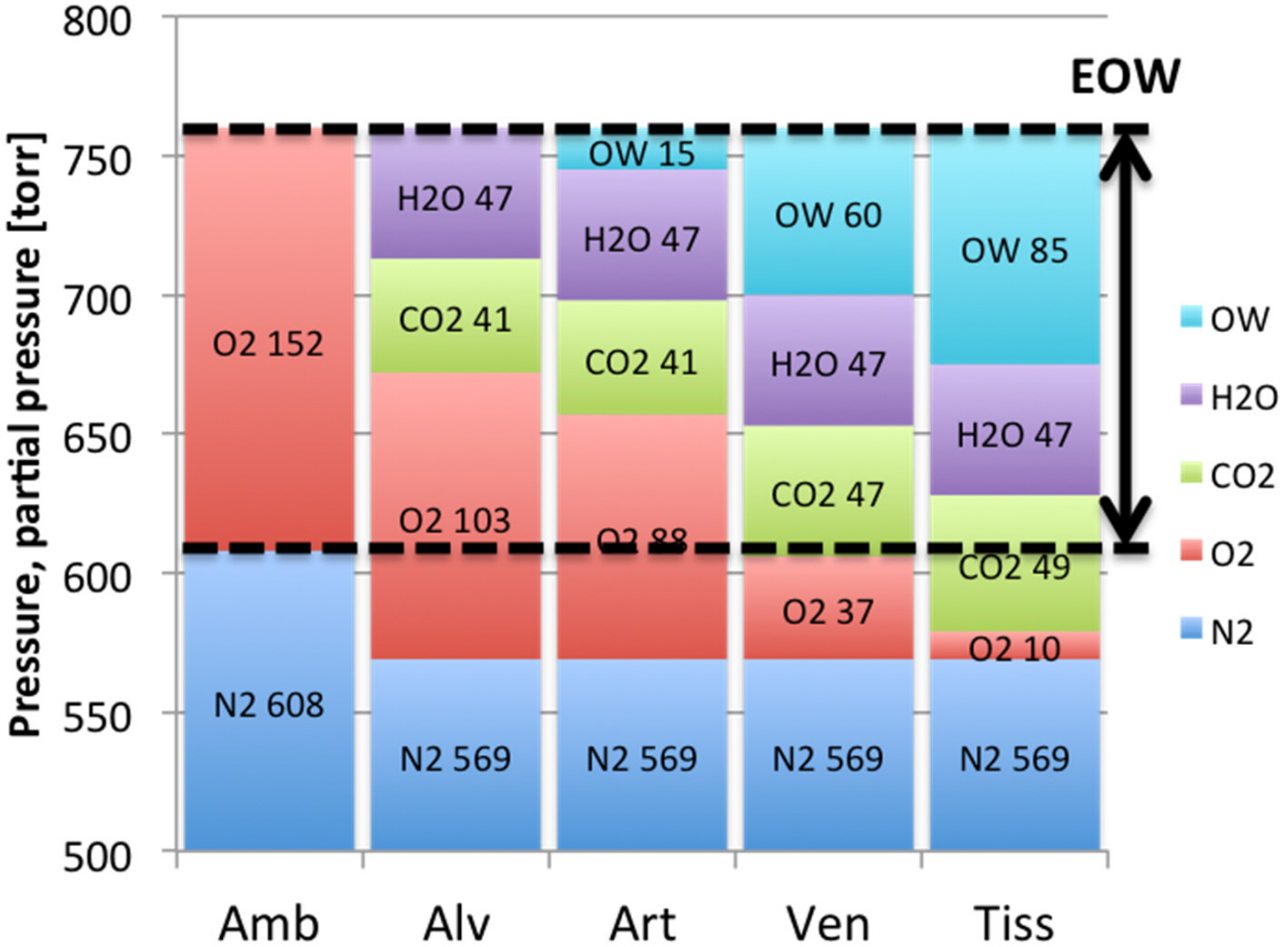
The Extended Oxygen Window concept. Standard oxygen window is about 60 torr in venous blood, and about 85 torr in tissues. When extended by part of partial pressure of carbon dioxide and water vapor, this value can be roughly equalized to partial pressure of oxygen in inspired breathing mixture.

It is now generally agreed that ΔP for saturation diving is related to oxygen, but in our model this relation goes even further, appointing oxygen as the only factor enabling safe desaturation of inert gas from the human body without creating gas bubbles. Oxygen is consumed in living tissues and this metabolism decreases partial pressure of oxygen in tissues. In return, carbon dioxide is produced; but due to its higher solubility, an increase in partial pressure of carbon dioxide in tissues (and returning venous blood) is of a lesser degree than a decrease in oxygen. Roughly speaking, an oxygen tension of 100 torr, as in arterial blood, corresponds to an approximate content of 3 ml O2 per 100 ml blood. Theoretically, if each oxygen molecule were exchanged during the metabolic process into a carbon dioxide molecule, there would be no change in the dissolved gas volume, but the tension would fall to less than 5 torr, only because carbon dioxide is more soluble than oxygen [16]. This phenomenon has been already described several times by different authors using different terms: partial pressure vacancy [17], inherent unsaturation [18] or oxygen window [19, 20]. Values for partial pressures of physiological gases (oxygen, carbon dioxide and nitrogen) and water vapor, as well as unsaturation created by metabolism are presented in Fig 1. When breathing air at normobaric conditions, the physiological unsaturation is about 60 torr and it increases proportionally with PiO2 at least up to PiO2 of 0.9 ata [21].

In our model, we assumed that, when speaking of transport of inert gas in this virtual space, this unsaturation could be safely extended by the water vapor tension (47 torr), which does not participate in the creation of gas bubbles (even if it joins the content of gas bubbles as a consequence of temperature), and at least part of partial pressure of carbon dioxide (45–49 torr), which is highly soluble and chemically active; it can be easily transported bound to hemoglobin and plasma proteins as well as converted to bicarbonate ions. Some elasticity of tissue cells keeping inert gas in a solution to some degree probably plays a role there as well. Therefore, in our model, we assumed that the oxygen window described originally by Behnke as 60 torr, can be safety extended by 90 torr, giving approximately 150 torr when breathing air at normobaric conditions, which equals to the inspired partial pressure of oxygen (PiO2). We call it the Extended Oxygen Window (EOW) concept.

The value of ^max^T1/2 is 360 minutes [14, 15]. This means that k values are kN2 = 1,925×10^-3^ hour-1 (which results in a decompression rate of 5.48 kPa/hrs for nitrox with PiO2 of 50 kPa). So the safe rate of saturation decompression can be easily calculated with Eq 1 using values of ΔP = EOW = PiO2 and T1/2 of 360 min.

But using the EOW concept implies also that at the beginning of the saturation, there is already some unsaturation (equal to the EOW), which can be passed at a faster rate as it does not induce any supersaturation. Theoretically, it should be possible to pass this phase in one step, assuming reasonable decompression rate, but—taking into account the primary assumption of physiological rationale—one can assume that the maximum allowable rate of decompression in this phase depends on the maximum allowable out flow of inert gas from fast compartments. Such values have never been measured in any real experiments concerning human saturation decompressions, except where measured during isobaric decompressions at atmospheric conditions (Pamb = 1 ata) when inert gas (nitrogen) was eliminated from human body during oxygen breathing [3]. We used those limits as the maximum allowable elimination out-flow of inert gas from fast compartments.

The maximum inert gas flow (IGF) from different compartments can be calculated using the modified 5-compartmental human body model described by Jones (Eq 7) [3]:

$$IGF=111\cdot e^{-0.462\cdot t}+193\cdot e^{-0.087\cdot t}+428\cdot e^{-0.024\cdot t}+95\cdot e^{-0.008\cdot t}+600\cdot e^{-0.002\cdot t}$$ (7)

The summary of compartments is presented in Table 1.

**Table 1:** Theoretical body compartments [3].

Compartment:	1	2	3	4	5
Volume* of tissue [L]	10.16	17.10	38.80	(-)**	16.80
Volume nitrogen [L]	0.111	0.193	0.428	0.095	0.600
Half-time [min]	1.5	8	29	87	360***
K	0.462	0.087	0.024	0.008	0.002
Max elimination rate**** [ml/min]	1.78	1.63	2.06	0.24	0.51

* Assuming negligible fat content for non-fat tissues.

** Presumably representative of intestinal gaseous nitrogen.

*** Original value of half-time for the slowest compartment found with isobaric decompression (277 min) was replaced with half-time for saturation diving (360 min).

**** Maximum elimination rate was calculated as the highest elimination rate during isobaric decompression.

**Fig 2:**
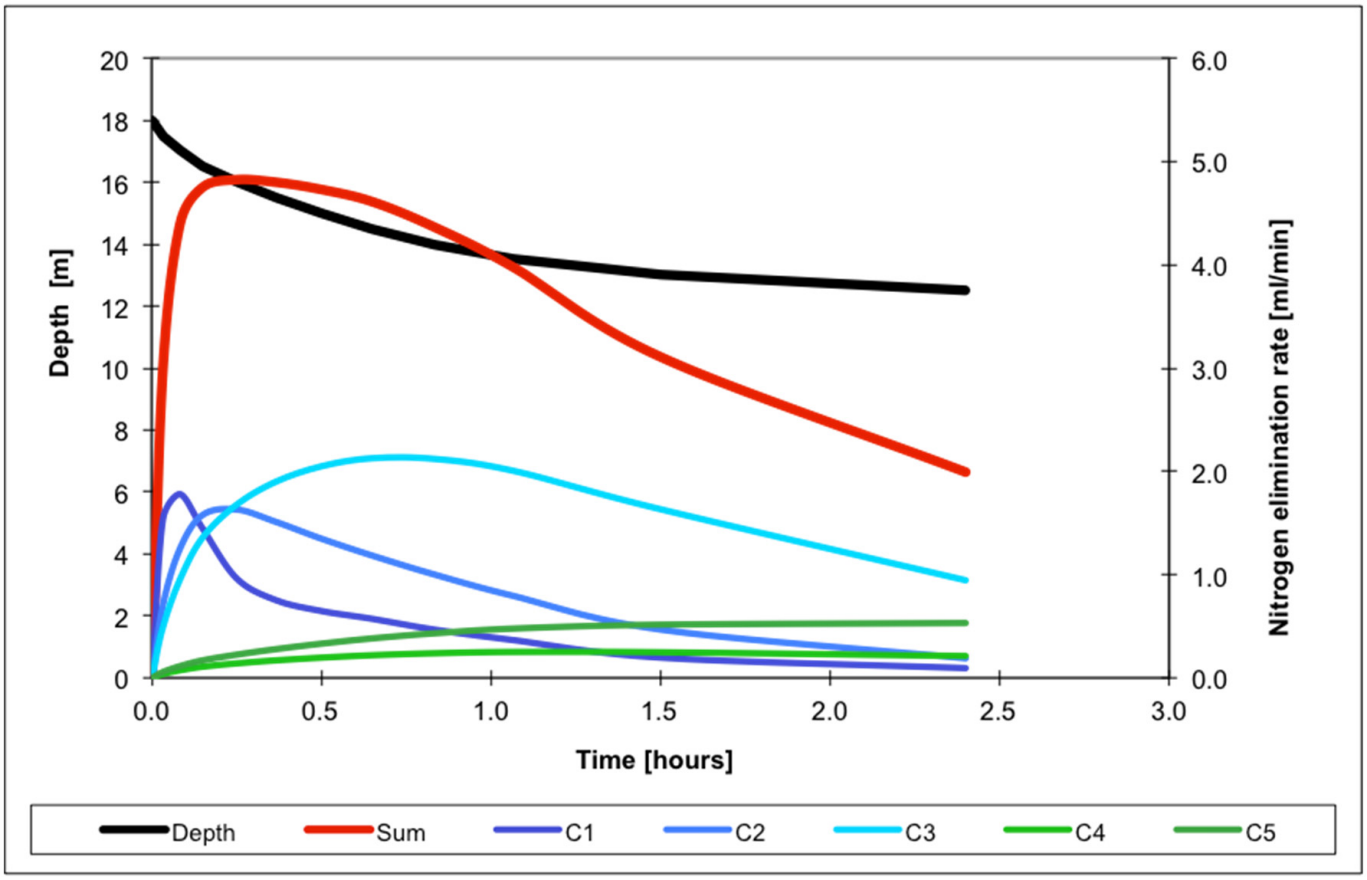
Nitrogen elimination rates calculated for different compartments during Phase 1 of nitrox decompression (PiO_2_ = 0.5 ata).

In order to conduct Phase 1 of saturation decompression, a decrease of ambient pressure was programmed in order to keep elimination rates of nitrogen from all compartments below the specified maximum (Fig 2, Table 2).

**Table 2:** Decompression rates during Phase 1 of saturation decompression.

Depth change	Time [min]	Decompression rate [m/min]
1^st^ 0.5 m step from saturation plateau	2	0.250
2^nd^ 0.5 m step from saturation plateau	3	0.167
3^rd^ 0.5 m step from saturation plateau	4	0.125
4^th^ 0.5 m step from saturation plateau	6	0.083
5^th^ 0.5 m step from saturation plateau	7	0.071
6^th^ 0.5 m step from saturation plateau	8	0.063
7^th^ 0.5 m step from saturation plateau	9	0.056
8^th^ 0.5 m step from saturation plateau	11	0.045
9^th^ 0.5 m step from saturation plateau	14	0.036
10^th^ 0.5 m step from saturation plateau	26	0.019
11^th^ 0.5 m step* from saturation plateau	54	0.009

* this step was used only for saturation decompression with compressed air (PiO_2_ = 0.588 ata), for other breathing mixtures, when PiO_2_ = 0.5 ata, rate of saturation decompression in this step was already as calculated in Phase 2 of decompression.

In order to confirm the correctness of the EOW model and its parameters (ΔP and T1/2), its parameters have been used for estimation of risk of DCS in published systems of saturation decompressions using air and nitrox, in the range from 7.7 to 50.3 meters, where DCS cases were observed (for references, see Table 3). We calculated the supersaturation exceeding the EOW using parameters of our model (ΔP = PiO2 and T1/2 = 360 min), noting how much time had been spent in each range of supersaturation (in steps of 10 kPa) (Table 3). **If there was no significant supersaturation, which means that driving pressure ΔP was kept below the EOW, or at least not exceeding EOW by more than 10 kPa, there was no single case of DCS reported. When any system allowed for supersaturation to occur, the DCS rate depended on how much supersaturation exceeded the EOW**.

**Table 3:** Calculation of supersaturation exceeding the extended oxygen window.

Nr	Depth[mH_2_O]	Deco time[min]	Number of bends	Number of divings	Percent of bends	Reference	Time [min] of SS* 0–10 kPa	Time [min] of SS* 10–20 kPa	Time[min] of SS* 20–30 kPa	Time[min] of SS* 30–40 kPa	Time[min] of SS*> 40 kPa
1	50.3	4281	4	10	40.0	[5]					495
2	8.9	2	4 (7)#	15	26.7 (46.7)#	[32]					73
3	40.2	3108	3	12	25.0	[40]				218	
4	7.7	2	0 (5)#	19	0.0 (26.3)#	[32]				90	
5	18.0	1269	4	10	40.0	[7]				45	
6	18.3	1198	2	23	8.7	[40]			720		
7	18.0	1629	3	10	30.0	[7]			60		
8	18.0	1800	1	20	5.0	[7]		1091			
9	30.0	2537				[62]		835			
10	18.0	1679	1	10	10	[7]		350			
11	20.0	1917				[62]		139			
12	40.2	3907	1	18	5.6	[40]		1416			
13	22.3	2019	1	24	4.2	[40]		1225			
14	18.0	2200	0	10	0.0	[7]		93			
15	18.0	1687	0	19	0.0	[63]	30				
16	30.0	2835	0	11	0.0	[64]	29				
17	45.0		0	24	0.0	[64]	30				
	**7.79÷50.3 m**		**32**	**235**	**13.6%**						

* SS—Supersaturation calculated as over pressure above the PiO2

After a preliminary evaluation of the EOW model, the correctness of the system had to be verified in prospective real saturation exposures. So the experimental aim of the study was to verify by saturation decompressions whether those parameters related to physiological values could be used for programming safe saturation decompressions using compressed air and nitrox in the whole range of depth available for those gases, namely from 18 to 45 meters.

## Material and Methods

**Fig 3:**
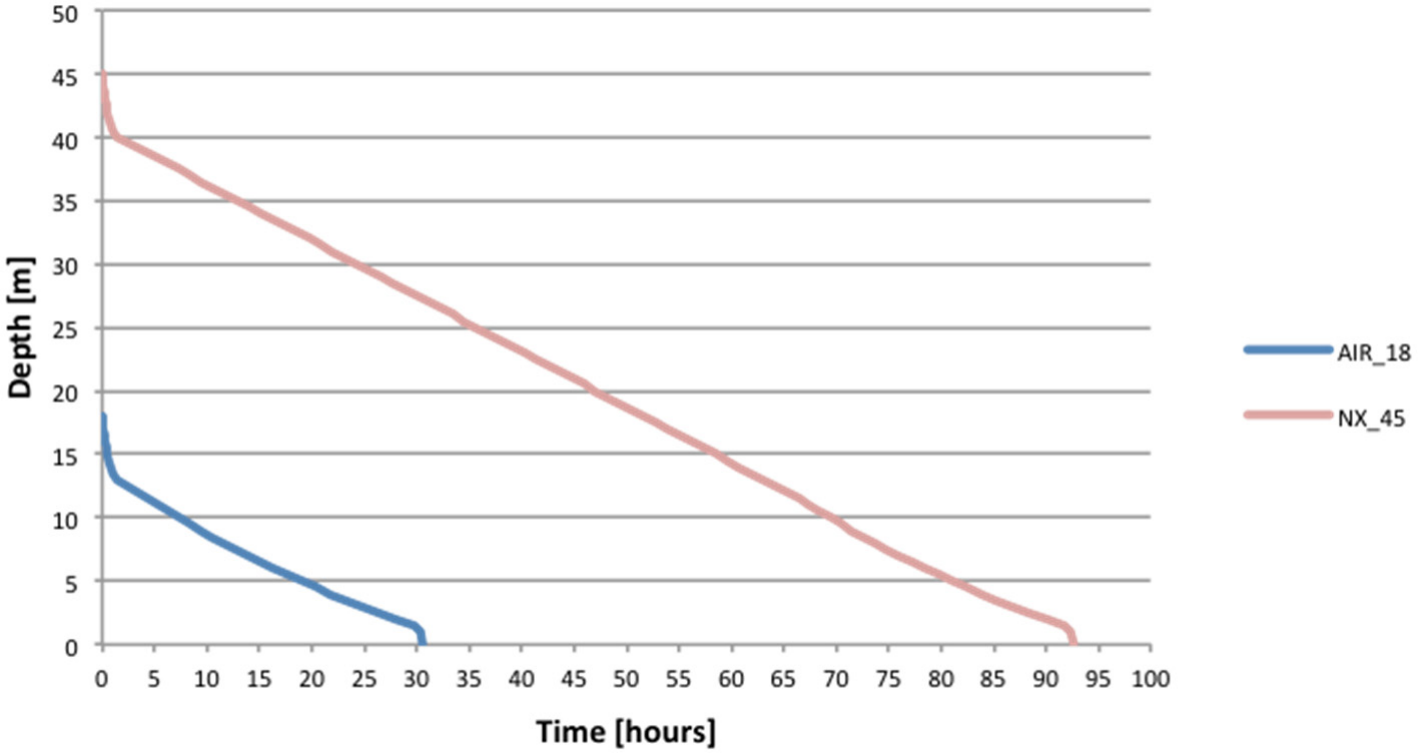
Example profiles of saturation decompressions created with the EOW concept. A typical shape is clearly visible for both compressed air (FiO_2_ = 0.21) from a depth of 18 m and nitrox (PiO_2_ = 0.4 ata) from a depth of 45 meters. The first phase of decreasing ambient pressure from plateau pressure through the EOW is relatively fast. Then the rate of decompression depends on PiO_2_, so for nitrox, this phase is linear with a constant rate; and for air, when oxygen partial pressure changes due to constant fraction of approximately 21%, the decompression rate decreases. In case of using nitrox, from about 11 meters of depth, the decompression rate also decreases, as a fractional amount of oxygen cannot exceed 24%. The very last phase of decompression (from about 1.5 meter to surface) is a fast decrease of ambient pressure in order to induce slight supersaturation (for details, see explanation in the text).

According to the model, a decompression profile defined by the EOW is divided into three phases (Fig 3).

Phase 1 is a fast initial ascent from saturation plateau through the EOW equal to PiO2 in breathing mixture. It is conducted with a decreasing rate in the time of 90 minutes when using nitrox with PiO2 of 50 kPa or 144 minutes when using compressed air (PiO2 of 56 kPa). The decompression rate changes as described in Table 2 according to maximum inert gas flow from compartments faster than the limiting one.

Phase 2 starts when EOW has been passed and the slowest compartment eliminating inert gas starts limiting the desaturation process. In this phase, the decompression rate is proportional to PiO2 with the proportionality coefficient related to the inert gas elimination factor from the limiting (slowest) compartment. For nitrox with PiO2 of 50 kPa, the rate of decompression is 5.48 kPa/hrs (0.548 m/hr, 0.009 m/min). When breathing compressed air, a fractional amount of oxygen is constant, but partial pressure changes, depending on ambient pressure and decompression rate changes, accordingly.

Moreover, when breathing compressed air, Phase 2 extends until end of decompression. Otherwise, Phase 3 of decompression starts at a depth of 11 meters, when fractional content of oxygen equals 24% (assuming 50 kPa partial pressure of oxygen). This fractional level should never be exceeded in a hyperbaric environment due to safety reasons in order to avoid fire risk. Therefore, in this phase, the partial pressure of oxygen gradually decreased in order to keep it at level of 23% (±1%). As a consequence, the decompression rate also gradually decreased proportionally to PiO2.

When pressure in a hyperbaric chamber was equal to 1.5 m, the decompression rate was manually modified in order to decrease ambient pressure to normobaric pressure in less than 60 minutes (regardless of the breathing mixture and calculated decompression rate). This procedure served as an internal control of correctness of the model and its parameters, as it was expected that slight supersaturation can be safely tolerated if created at the last moment of decompression. If there were any gas bubbles created at the beginning of decompression, such supersaturation would induce symptoms of DCS. This approach was for research purpose only. In standard operations, the calculated decompression rate can be extended up to surface (normobaric pressure).

The study protocol was approved by the Ethics Committee of the Medical University of Gdansk and—according to this protocol—all participants provided written informed consent for participation. Young, healthy volunteers (either recreational or professional divers) aged from 20 to 44 years were chosen to participate in the experiments. The exclusion criteria included any chronic disease, and history of decompression disease or injury, which could provoke decompression illness—for example, bone fractures. Some divers had been preliminarny trained in a hyperbaric environment, and some had not.

Expositions were made in a dry three-chamber diving simulator (LSH-200) located in the National Center for Hyperbaric Medicine in Gdynia, Poland. The system can be pressurized up to 200 m with any breathing mixture of oxygen, nitrogen and helium. Environmental parameters are controlled by the computerized automatic life-support system—ambient pressure: ±0.1%; partial pressure of oxygen: ±1%; fractional amount of carbon dioxide: ≤0.3% in samples decompressed to normobaric pressure; temperature: 23–25°C; relative humidity: 40–70%. In every single exposure exposition, two to five diver-testers took part. While staying on the saturation plateau, partial pressure of oxygen in the chamber was kept on a constant level of 40 kPa (± 2.5 kPa), and the content of the atmosphere was controlled by the chemical analysis of decompressed gas samples using the HPLC for any toxic impurities. Every day divers were given a task of 30-minute work on a cycloergometer (100–150 watts) avoiding any significant exercise at least 12 hours before commencing saturation decompression. The shortest stay under pressure was 2.5 days (60 hours) to ensure full saturation with nitrogen.

After 2.5–4 days of staying on a plateau, the partial pressure of oxygen in the chamber was increased from 40 kPa to 50 kPa (± 2 kPa), and after 3 hours, a continuous decompression from saturation plateau to surface pressure was commenced using the decompression profile calculated for specific breathing gas of decompression as described above. Due to safety reason of fire prevention, the maximum allowed fractional content of oxygen in the chamber atmosphere was 24% (23 ±1%).

After every exposure, divers were tested by a physician responsible for experiments and stayed near the chamber for 24 hours. The decompression was assumed safe unless one or more of the following occured: 1) bends, 2) physiological disturbances, 3) subjective complaints, and 4) abnormal results of basic laboratory blood tests (morphology, platelet counts, liver tests). After 24 hours divers were dismissed but requested to report any symptoms suggesting DCS directly to the researchers. They were also contacted after one week for any late occurring symptoms.

## Results and Discussion

**Fig 4:**
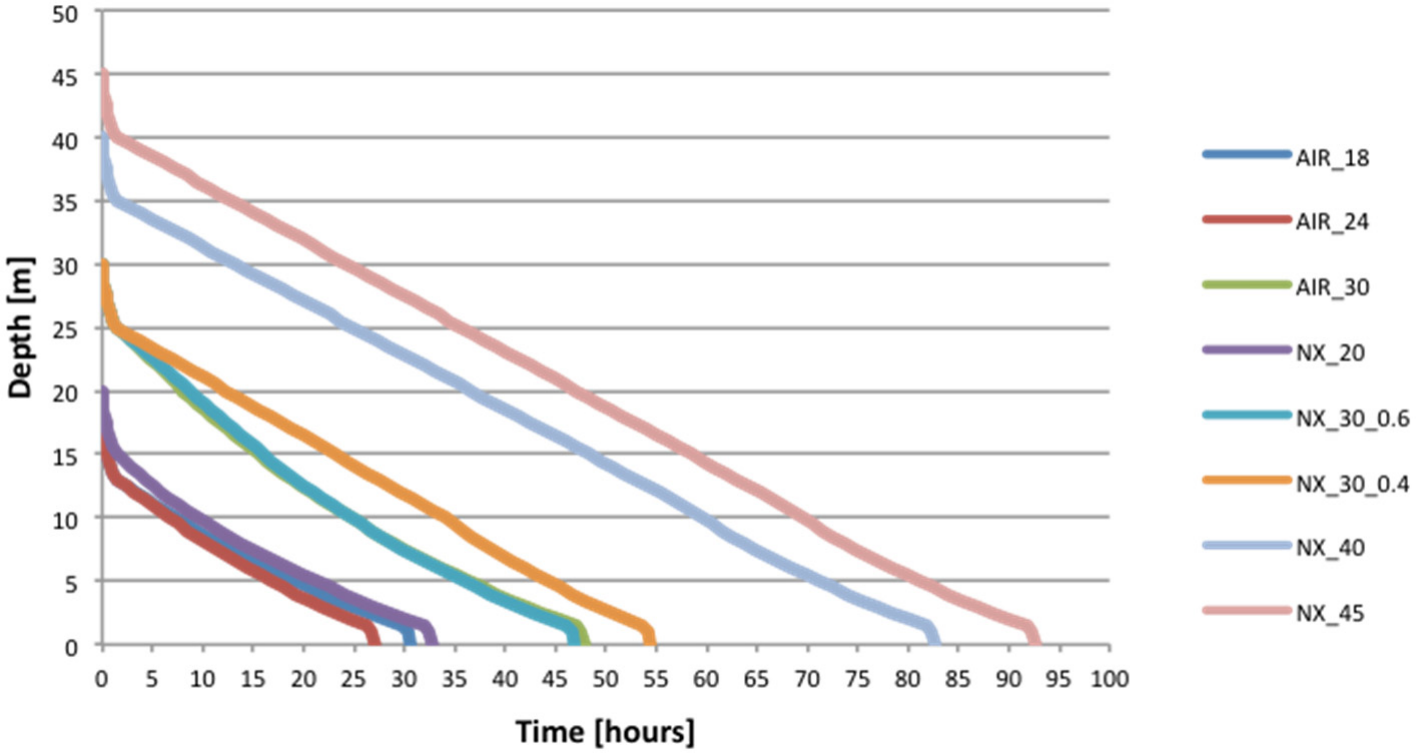
Profiles of decompressions planned and conducted using the EOW concept.

In total, 72 man-expositions were conducted during the study, including 36 man-expositions using compressed air in a range of depth from 18 to 30 m and 36 man-expositions using nitrox (nitrogen and oxygen) in a range of depth from 20 to 45 m. All saturation decompressions are presented in Fig 4 and Table 4.

**Table 4:** List of saturation exposures.

Breathing gas	Number of man-expositions	Depth	Time at plateau (in total)	Decompression time (in total)
Air	36	18 m– 30 m	1,932 hrs = 80.5 days	1,336 hours = 55.7 days
Nitrox	36	20 m– 45 m	2,640 hrs = 110 days	2,092.4 hours = 87.2 days
**TOTAL:**	**72**		**4,572 hrs = 190.5 days**	**3,428.4 hours = 142.85 days**

Neither bends nor any other symptoms of decompression stress were observed in any of the 72 expositions. Statistical analysis of obtained results based on a deterministic criterion of the lack of bend symptoms suggested that the probability of occurrence of bends in the employed decompression method does not exceed 0.042 with upper confidence interval of 95%, which means that the upper 95% CI for DCS is less than 4.2% (calculations made by using the rule of three, as there was no positive event [22]).

During real exposures with humans, we confirmed that use of physiological parameters based on the Extended Oxygen Window concept (ΔP = PiO2, T1/2 = 360min) for saturation decompressions using compressed air and nitrox in the range of depth from 18 to 45 meters allows safe decompression after very long exposures.

The maximum allowable gradient for elimination of inert gas ΔP after saturation exposure has been studied extensively for compressed air in the past. Many different relations between plateau pressure P1 (called also “storage depth” in operational procedures) and maximum allowable reduction of pressure ΔP after saturation stay at plateau pressure P1 have been proposed (Table 5).

**Table 5:** ΔP for air saturations.

YEAR	AUTHOR	REFERENCE	RELATION ΔP(P_1_)	DD (m)	ΔD^18^ (m)
1908	Boycott	[2]	**ΔP** = 0.500×P_1_	10.0	14.0
1956	Des Grange	[65]	**ΔP** = 0.341×P_1_ + 0.303	9.8	12.6
1976	Bernard	[66]	**ΔP** = 0.284×P_1_ + 0.408	9.7	12.0
1969	Hempleman	[67]	**ΔP** = 0.268×P_1_ + 0.410	9.3	11.6
1979	Yount	[18, 68]	**ΔP** = 0.289×P_1_ + 0.355	9.1	11.6
1965	Workman	[69]	**ΔP** = 0.273×P_1_ + 0.378	9.0	11.4
1969	Buhlman	[70]	**ΔP** = 0.286×P_1_ + 0.335	8.7	11.6
1971	Stubbs	[71]	**ΔP** = 0.281×P_1_ + 0.302	8.1	10.9
1935	Hawkins	[71]	**ΔP** = 0.444×P_1_	8.0	12.4
1973	Nishi & Kuehn	[72]	**ΔP** = 0.278×P_1_ + 0.303	8.0	10.8
1937	Yarbrough	[71]	**ΔP** = 0.429×P_1_	7.5	12.0
1986	Yount (human)	[68]	**ΔP** = 0.271×P_1_ + 0.245	7.1	10.1
1977	Hennessy	[36]	**ΔP** = 0.265×P_1_ +0.250	7.0	9.9
1965	Zal’tsman	[73, 74]	**ΔP** = 0.387×P_1_	6.3	10.8
2000	Polish DSK system	[23]	**ΔP** = 0.200×P_1_	2.5	5.6
1967	Behnke	[19]	**ΔP** = 0.200×P_1_ - 0.120	1.0	4.4
1967	Griffiths (for caisson workers)	[75]	**ΔP** = 0.500×P_1_ - 0.500	0.0	9.0

The value of DD (Direct Decompression) is defined as the depth of saturation (equivalent to plateau pressure P_1_) after which diver can be surfaced without need of any decompression other then reduction of pressure in few minutes only; the value of ΔD^18^ describes maximum allowable reduction of depth/pressure after saturation at depth of 18 m (equivalent to pressure of 2.8 ata). Systems are ordered by decreasing value of DD and ΔD^18^.

The first relation, already suggested by Haldane [2], ΔP = 0.500×P1 is a mathematical equation describing his assumption that one can safely reduce pressure by half even after saturation stay at depth. It suggests that it is possible to surface directly after an infinite stay at a depth of 10 meters when breathing air and a fast ascent for the first 14 meters after saturation with air at 18 meters. No one has ever tried this, but later experiments from even shallower depths induced many cases of DCS. The last equation in the table (ΔP = 0.500×P1-0.500) was proposed in 1967 by Griffiths for caisson workers as a modification of Haldane’s assumption, but applied to relative pressure read-outs from manometers scaled in gauge pressure. In fact, this is an example of the introduction of experimentally found safety factors to increase safety of decompression by decreasing pressure reduction. Interestingly, two systems, one used in Poland and the other one described theoretically by Behnke, use equations which give significantly smaller values than others both for DD (2.5 and 1.0 m, respectively) and ΔD18 (5.6 and 4.4 m, respectively) [19, 23]. If all relations were expressed in the general form of ΔP = a×P1 + b, only those two equations would show direct connection with oxygen fraction in inspired breathing mixture (FiO2 = 0.2). These relations will be also be discussed later on.

The other parameter, which limits the desaturation process, depends on the elimination rate of inert gas from the slowest compartment, and is described in Eqs 1 and 2 as the maximum half-time (^max^T1/2) for elimination of nitrogen. Since 1908 when J. S. Haldane proposed 75 min for the slowest compartment based on animal research using goats, many longer values have been proposed in the literature. The evaluation of the ^max^T1/2 for nitrogen is presented in the following table (Table 6). It is noteworthy that these values have been used exclusively for mathematical calculations, so it was never discussed which anatomical tissue limits saturation.

**Table 6:** Maximum elimination half-time for nitrogen.

YEAR, AUTHOR, REF	^max^T_1/2_
1908, Boycott, Damant & Haldane [2]	75
1937, Yarborough [24]	75
1935, Behnke [25]	81.5
1937, Behnke [20]	85
1944, Smith & Morales [76]	87
1950, Van Der Aue [25]	120
1956, Des Granges [77]	120
1967, Behnke [19]	120
1944, Behnke [78]	138
1950, Jones [3]	147
1950, Bateman [25]	207
2004, Flook [79]	211.3
1964, Workman [25]	240
1989, Wienke & Hills [80]	240
1950, Jones [3]	277
1997, Gerth & Vann [81]	317–326
1967, Schreiner & Kelley [82, 83]	320
1992, Parker [81]	338
1983, Doboszynski & Lokucijewski [14]	360
1989, Wienke [84, 85]	360
1989, Wienke [84, 85]	360
1992, Conkin [29, 30]	360
1996, Sicko & Kot [15]	360
2012, Blatteau [59]	400
1967, Buhlman [86, 87]	420–480
1997, Thalmann [31]	420–487
1973, Hamilton [25]	480
1990, Wienke [27, 88]	480
1983, Buhlmann [26]	635
1966, Schreiner [25]	720
1977, Gulyar [89]	720
1990, Wienke [27, 28]	720
1980, Miller [25]	1280

Rows are ordered by increasing value of T_1/2_.

Based on animal research, Haldane originally proposed 75 min for divers, the smallest value in the table [2]. This value was also used almost 30 years later [24]. In the 1950s the longest half-time—about 270 min—was established during isobaric desaturation of inert gas while breathing pure oxygen [3], but a similar value—240 min—was proposed and has been used for decades in the US Navy [25], based on bounce (non-saturation) dives. At the same time others proposed values between 120 and 720 min. Later on, in the 1980s, significantly longer values were proposed, even 635 or 720 min [25–28], just to compensate high values accepted at the same time for ΔP. The longest value—1280 min—was proposed by Miller just for mathematical calculations [25], as there is no reason to expect that in human physiology there is any tissue of such slow perfusion, which could play the limiting role for saturation decompression. After decades of trying different values, several authors using different approaches including hypobaric exposures [29, 30], long saturation decompressions [15], and probabilistic decompression model risk predictions using linear-exponential kinetics [31] amicably report that physiological values of T1/2 (even if the specific anatomical compartment has not been identified) are somewhere between 320 and 420 min.

As can be seen from the above, the range of ^max^ΔP’s (from ΔP = 0.500×P1 to ΔP = 0.500×P1-0.5), ^max^T1/2’s (from 75 min to 1280 min) and the resulting value of k (from 0.009242 to 0.000541, respectively) is very wide. So, theoretically, the combination of both ΔP and ^max^T1/2 as describing the decompression rate using Eq 1 is even wider. But, from a physiological point of view, such situation is highly improbable, as there must be one set of limiting values for both parameters for describing a real human desaturation process after saturation.

Therefore, we used the EOW concept based on the physiological oxygen window created by metabolism. There is strong physiological rationale behind using this concept for defining the ΔP parameter, which has been already presented in the introduction, and our real saturations directly confirmed the concept. But there is also other evidence supporting this model and its parameters, including the dose-reaction relationship between the supersaturation exceeding the EOW and reported observations of gas bubbles found during direct decompressions after shallow saturations with air [32–34].

**Fig 5:**
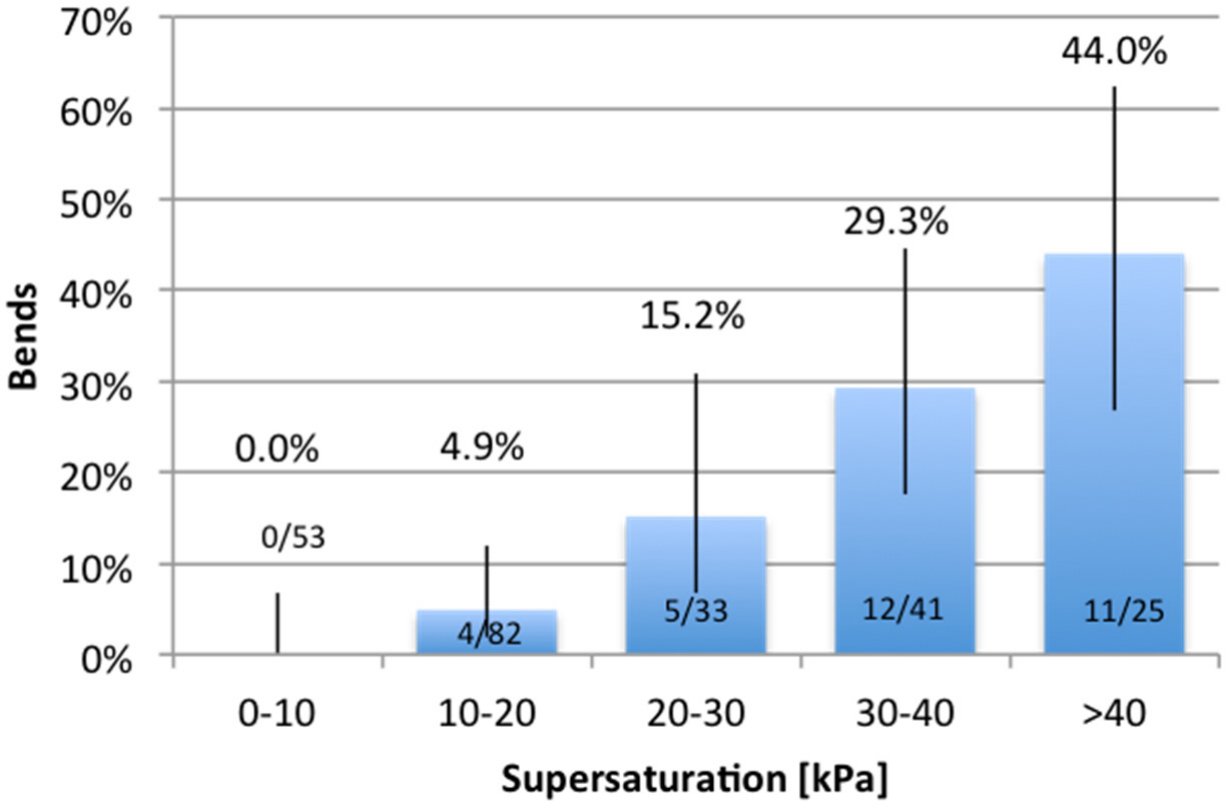
Dose-reaction between supersaturation exceeding the EOW for air and nitrox saturation decompressions published in literature (bars represent the 95% confidence interval).

The dose–response relationship, or exposure–response relationship, describes the change in effect on an organism caused by differing levels of exposure (or doses) to a stressor (overpressure/supersaturation) after a certain exposure time. When we applied the parameters used in our model against those air saturation decompressions reported in literature in the range between 7.7 and 50.3 m, we found the clear dose-response relationship between supersaturation exceeding the extended oxygen window (in steps of 10 kPa) with the rate of decompression sickness (Fig 5). If the supersaturation is kept close to zero level when compared to EOW (in the range of 0 to 10 kPa of supersaturation), there was no single case of DCS reported. In those systems where high supersaturation occurred (exceeding 40 kPa), DCS was observed in as many as 44% of the subjects, even if time spent with such supersaturation was sometimes relatively short (slightly more than one hour). **Having such a clear relation between supersaturation exceeding the EOW with rate of DCS means that our model can be used not only for programming of saturation decompressions, but also for estimation of DCS risk for any other saturation systems.**

Other indirect evidence that supports our model comes from observations of gas bubbles found during direct decompressions after shallow saturations with air. Direct decompression is defined as the depth of saturation after which the diver can surface without need of any decompression other than the reduction of pressure in a few minutes only. It is expected that this value represents the largest overpressure in tissues that can be sustained by a diver. In the past, this value has been used to estimate the driving force for desaturation of inert gas (ΔP) (see Table 5). Taking into account our model, the maximum allowable depth after which divers can conduct the direct decompression to surface after infinite stay at pressure breathing air (FiO2 = 0.21) is only 2.5 meters (ΔP = PiO2). This limit is significantly less than values accepted in other systems. However, there are some reports in the literature that gas bubbles have been observed after direct decompressions to surface after saturation with air at about 8.7–9.0 m (50% DCS, 7 out of 15), 25% DCS (5 out of 19) from depth of 7.7–8.0 m and 16% DCS (22 of 138) deeper than 6 meters [32–34]. Even if no DCS was observed during direct decompressions from depths shallower than 6 m (0 out of 448) [35], venous gas emboli (VGE) were noted after direct decompressions from depths as shallow as 6.4, 5.0, and even from 3.8 m (!) [33]. In our model we assumed even more restrictive limitations (2.5 m when breathing air). In the literature, there are two systems described by Behnke and Griffiths that suggest even lower values, 1.0 m and 0.0 m, respectively [19, 36]. As mentioned previously, the last equation in the table is the mathematical representation of the practical approach of Griffiths for decompressing caisson workers using the modified Haldane rule (dividing pressure by a factor of 2), but applied to relative pressure read-outs from manometers scaled in gauge pressure instead using absolute pressure as suggested by Haldane. Such a technique gave safer results. For example, divers from a depth of 18 meters could be decompressed to a depth of 9 meters only by Griffiths, which is deeper (and which means safer), then by 14 meters to a depth of 4 meters only, as allowed by Haldane’s equation (see the column D18 of Table 5). In fact, Griffiths’ approach is an example of the introduction of an experimentally found safety factor to increase safety of decompression just by decreasing pressure reduction. Due to its practical approach, it cannot be considered as valuable information for a description of the physiological processes behind it.

The other limit for direct decompression, which is lower than this based on EOW, comes originally from Behnke and his first description of the oxygen window [19]. His equation leads to the declaration that direct decompression can be safely conducted only from a depth of 1.0 meter. Most certainly, it is perfectly safe from a practical point of view. But yet another consequence of the Behnke’s relation is that from the air saturation depth of 18 meters, one could decompress divers only by 4.4 meters in Phase 1 of decompression. During our experiments, we decompressed divers from a depth of 18 meters up to 12.5 meters passing the EOW equivalent to a depth change of more than 5.5 meters. If the limit suggested by Behnke were true, the supersaturation created at the beginning of long decompression lasting more than 30 hours almost surely would induce gas bubbles and DCS, at least in some divers. **Therefore, it seems that values of ΔP** **lower** **than PiO_2_ are unnecessarily too restrictive, and values** **greater** **than PiO_2_ induce gas bubbles.**

In fact, the concept that the decompression rate after saturation is directly proportional to partial pressure of oxygen (PiO2) has been proposed several times in the past [37–45]. However, it has never been directly related with the physiological concept of the EOW but rather on simple proportionality of the decompression rate to PiO2 with the k coefficient treated as a mathematical parameter only, not representation of the limiting body compartment. As a consequence, according to the best knowledge of authors, **the assumption that ΔP equals PiO_2_ as a consequence of the EOW has never been implemented explicitly in any existing system for saturation decompression**. Even in the US Navy, when Vann proposed a similar theoretical model, he recommended reducing the ascent rate for deeper saturation depths, which is in clear disagreement with the model driven by oxygen when the decompression rate should be independent from depth [38]. Interestingly, the systems published by the US Navy went in an opposite direction, allowing faster ascent rates for deeper saturation depths [4]. It is evident that none of the existing systems require or suggest really fast ascent in the first phase of decompression, namely through the EOW, just using the constant rate of decompression [4, 11, 44, 46–49].

**Fig 6:**
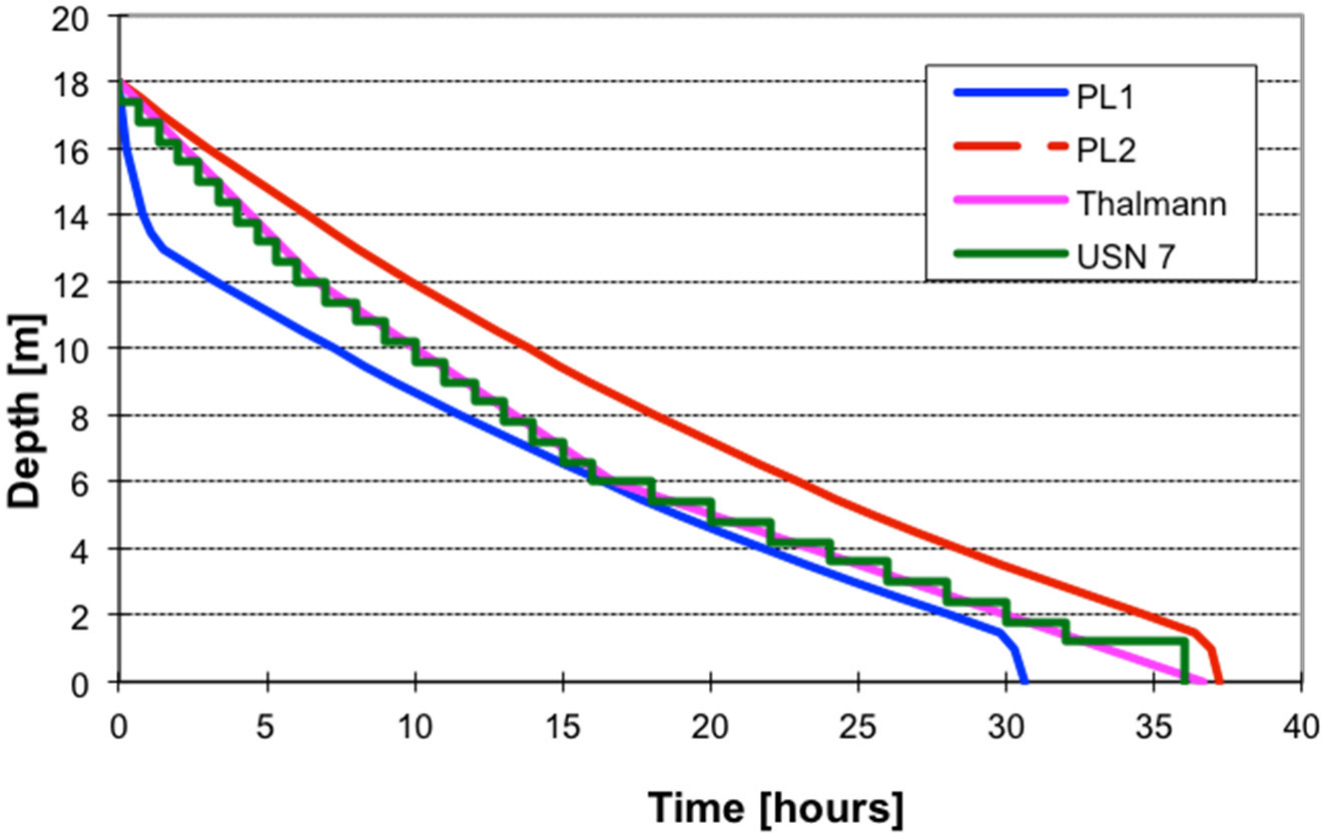
Comparison of different saturation decompression profiles. PL1 is the real conducted air saturation using the EOW concept; PL2 is a hypothetical decompression profile after air saturation without fast Phase 1. Thalmann and US Navy T7 show air saturation decompression proposed in the US Navy [4, 7].

**Fig 7:**
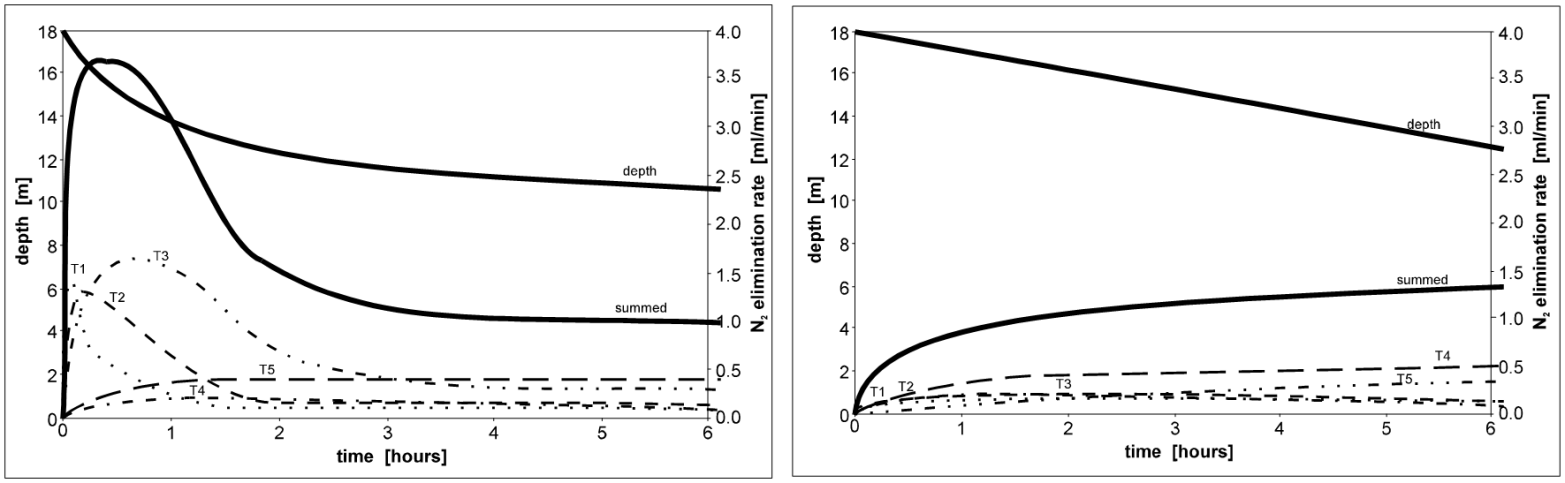
Comparison of nitrogen elimination rates in different saturation systems. The left graph shows fast initial phase of saturation decompression indicated by the EOW, and the right graph shows saturation decompression during which the decompression rate is constantly slow from the beginning (US Navy).

The easiest way to recognize whether a system relies fully on the EOW concept is the shape of the decompression profile, especially Phase 1, which is a relatively fast decrease of pressure when passing an inherent unsaturation according to the EOW (Fig 3). As a direct consequence of this acceleration, the difference in decompression times between different systems can be significant (about 20% in case of air saturation at 18 meters of depth) (Fig 6). Theoretically, if one would decompress without acceleration within Phase 1, decompression afterwards at saturation at 18 meters of depth would be longer by more than 20% (6.6 hrs/30.6 hrs). Hypothetically, we cannot exclude that this fast ascent in the first stage of decompression eliminates such a large amount of inert gas dissolved in tissues faster than from the limiting compartment that it creates space for the elimination of inert gas from the limiting compartment in Phase 2 (Fig 7). If such a phenomenon occurs this would mean that Phase 1 not only increases efficiency of decompression, but also increases safety. This has not been proven directly in our experiments, but the absence of DCS symptoms after many hours of later continuous decompression suggests that indeed, this technique does not induce a significant amount of free gas phase at the beginning of decompression.

Our study has two main limitations. One limitation is the lack of a direct search for gas bubbles using Doppler monitoring of decompressed divers. Therefore, the main confirmation is based on clinical evaluation of DCS occurrence, and from dose-reaction relationship for those experiments where there were DCS cases. Such an approach allows us to compare our system with other decompression schedules validated using same criteria, but precludes drawing final conclusions concerning the level of decompression stress induced by silent gas bubbles, if any. There are also other indicators proposed as markers of decompression stress after surfacing, including parameters of activation of platelets and fibrinolysis, but none of them was generally approved as a discriminating factor [50–52]. Another limitation is that we validated our model only in a physiological range of PiO2 (from 0.4 to 0.6 ata). It can be assumed that in this range, both the oxygen window and the EOW are directly proportional to PiO2 [21, 53]. This range is most often used in operational practice, as it does not induce any significant vasoconstriction or toxic effects on lungs or CNS, so it can be kept for months [54]. However, it would be interesting to know how the model behaves outside this range of partial pressure of oxygen. Such knowledge could be of great value also for practical use of the model for planning of saturation decompressions in case of emergency, when high partial pressure of oxygen is empirically used for accelerating desaturation. Some modeling research has already been done [55], some studies on animals have been conducted [56], several procedures have been recommended [57, 58], and recently, this method has been investigated in humans [11, 59]. Even if “the advantages of oxygen appear far less than predicted by current decompression models” [60], using a high amount of oxygen for accelerating decompression is already recommended in case of emergency [55, 61].

In summary, decompression after saturation with nitrogen based breathing mixtures is driven by oxygen. Initially, ambient pressure can be reduced at a higher rate allowing elimination of inert gas from faster compartments using unsaturation created by oxygen metabolism and properties of carbon dioxide and water vapor (the Extended Oxygen Window concept). Then, keeping the driving force (ΔP) for long decompression not exceeding partial pressure of oxygen in inspired breathing mixture (PiO2) allows optimal elimination from the limiting compartment with a half-time of 360 min.
